# The development of nursing-sensitive indicators: A critical discussion

**DOI:** 10.1016/j.ijnsa.2024.100227

**Published:** 2024-07-24

**Authors:** Edel Gormley, Michael Connolly, Mary Ryder

**Affiliations:** a University College Dublin (UCD) School of Nursing, Midwifery and Health Systems, University College Dublin, Dublin, Ireland; b Education & Research Centre Our Lady's Hospice & Care Services, Dublin, Ireland

**Keywords:** Nursing, Nursing indicators, Nursing care, Nurse's role, Quality, Values

## Abstract

**Discussion arguments:**

In a science-based profession, nurses must continuously monitor and evaluate the effectiveness of their care. However, data on what constitutes nursing care in practice and the delivery process is lacking. Insufficient evidence on how nurses contribute to patient care hampers the evaluation of nursing practice.

We discuss nursing-sensitive indicators, their origins, current applications, and challenges related to their use in evaluating the quality of nursing care. We analyse nursing-sensitive indicators in the context of criticisms levelled at the profession related to the lack of evidence to support their value in the larger healthcare environment.

**Conclusions:**

We have a disjointed approach to evaluating nursing care. Current systems designed to monitor nursing care, such as metrics and data sets, are not adequate or effective for comprehensively evaluating nursing care, considering the fundamentals and values of the nursing profession.

**What is already known about the topic**

• Robust evidence on the nursing contribution to patient care is scarce; part of the reason for this is flaws in the current methods of evaluating nursing care.
• Evaluation of care is necessary to demonstrate that it is effectively delivered and desired outcomes are achieved.
• There is a recognised need to better evaluate nursing care both nationally and internationally.

Alt-text: Unlabelled box

**What this paper adds**

• We provide a compelling synopsis of why current approaches to nursing care evaluation are not effective in practice.
• We acknowledge that as evidence-based practitioners, there is an urgent need for the development of evidence-based nursing indicators to comprehensively evaluate the nursing role in patient care.

Alt-text: Unlabelled box

## 1 Introduction

Nursing-sensitive indicators have been established as a means of monitoring nursing care quality (Alexander, 2007), they are described as the criteria for change in a person's health status that can be attributed to nursing care (Oner et al., 2021). There is strong evidence to suggest that nursing care makes a considerable contribution to the well-being of a patient (Jarelnape *et al*., 2023). However, there has been increased pressure on nurses to demonstrate in a measurable way the significance of their role (Visintini and Palses, 2023). As the largest segment of healthcare workers globally, nurses and their contribution to healthcare have been the focus of much speculation (World Health Organisation, 2020). Unfortunately, some of this attention to nursing practice has been brought about by negative reports of nursing care (Ball et al., 2018; Garling, 2008; Francis, 2013). In response, policy makers and healthcare systems have become more interested in how nursing care can be measured/ evaluated to ensure patient outcomes are being achieved (Muntlin, 2018).

In Ireland, nurses are required to be accountable for their work and obligated through their code of conduct to demonstrate the effectiveness of care delivered (Nursing and Midwifery Board of Ireland, 2021). There is an onus on nurses to utilise evidence-based practice. The Health Service Executive has recognised the importance of evaluating the unique contribution of nurses to patient care and has set about identifying specific and measurable elements of nursing care provided, which is necessary to ensure accountability and provide quality assurance to the public (Department of Health, 2017). This will also justify investment in nurse staffing and nursing services (WHO, 2020). Unfortunately, quality nursing care has been described as an ‘elusive concept’ (Griffith *et al*., 2008), recognising that measuring nursing care accurately is a challenging task. One of the greatest challenges is translating the complex role of nurses into measurable data, encapsulating their ability to achieve scientific evidence-based outcomes through care that upholds the fundamentals and values underpinning nursing practice (Moran, Bailey and Doody, 2021).

When reading about the measurement of nursing care in the literature, one finds that researchers have taken multiple approaches (Audet *et al*., 2018; Myers et al.*,* 2018; Driscoll et al.*,* 2018). Not all researchers use a conceptual or theoretical framework to guide their studies, but amongst those that do use a framework, the Donabedian model (Donabedian, 1988) is the most prominent conceptual framework used (Afeneh, Abu-Moghli and Ahmad, 2021). Nursing-sensitive indicators are increasingly adopted as a valid and reliable tool for measuring nursing care due to their potential to produce objective data (Oner *et al*., 2021). The Donabedian model is based on a substantially-linear point of view which assumes that structures influence processes and thus affect outcomes (Donabedian, 1988). However, caution must be exercised when using nursing-sensitive indicators. Despite a long history and many attempts to distinguish, develop, and standardise nursing-sensitive indicators, their use in healthcare remains problematic. Evidence available for nursing-sensitive indicators is scant and often vague due to variances in measurements used and lack of standardised definitions (Burston, Chaboyer and Gillespie, 2014; Afeneh, Abu- Moghli and Ahmad, 2021).

## 2 Background

The need to demonstrate the impact of nursing care on patient outcomes is not new. As early as the 1860s, Florence Nightingale compiled statistical records of patient outcomes in terms of whether patients were dead, relieved, or unrelieved (Montalvo, 2007). Nursing- sensitive indicator is a term that has been in use since the 1990s. Since that time, there have been many attempts to conceptualise it in a meaningful way for use in nursing practice (Dubois *et al*., 2017). Unfortunately, there remains a lot of uncertainty and confusion surrounding what constitutes nursing-sensitive indicators. Reading the literature around nursing-sensitive indicators can be overwhelming and lead to further ambiguity as opposed to clarity (Afaneh, Ahmad and Abu-Moghli, 2021). Understanding the evolution of nursing-sensitive indicators is helpful in understanding their current use in healthcare.

### 2.1 Development of nursing- sensitive outcomes

In the United States of America (USA) in the 1970s and 1980s, the United States Department of Health and Human Services came under pressure to evaluate the quality of healthcare services, as costs were soaring, and issues, such as limited access to healthcare and fragmented delivery of care, were causing public dissatisfaction and complaints (Gallagher and Rowell, 2003). While emphasis was placed on improving quality of patient care, it is important to note that there was also motivation to reduce the total cost per patient case, decrease patient length of hospital stay, and increase patient turnover (Girard, 1994). The context of this is relevant when considering the types of nursing outcomes originally investigated, bearing in mind that many of these outcomes are still being classified as nursing-sensitive outcomes today (Oner et al.*,* 2021).

Maas, Johnson, and Morehead (1996) used the term “nursing-sensitive indicators” when reporting on patient outcomes related to nursing practice. It was thought that this term was broad enough to encapsulate nursing structures and processes that influenced patient outcomes, either directly or indirectly. Structural indicators refer to factors that affect the context in which care is being delivered, such as the physical facility, staff numbers, skill mix, equipment, resources, and similar. Process indicators refer to transactions between patients and care providers and the technical delivery of care. Outcome indicators refer to endpoints of care, such as improvement in function, recovery, or survival; outcomes measurements seek to clarify if the goals of patient care were achieved (Haj, Lamrini and Rais, 2013).

The American Nurses Association adopted nursing-sensitive indicators as a means of describing all nursing aspects that influenced patient care using the Donabedian model to demonstrate nursing structures, processes, and subsequent outcomes (Dubois *et al*., 2013). The American Nurses Association started to try to identify relevant nursing-sensitive indicators to be examined further for their contribution to care. As a preliminary step, it began to investigate the numbers and mix of nursing staff along with nursing staff qualifications and their effect on patient outcomes. The Delphi approach was used to achieve consensus amongst a panel of nursing experts on what indicators should be selected as nursing quality indicators (Gallagher and Rowell, 2003). The outcome of the Delphi resulted in the selection of 10 nursing-sensitive indicators by the expert panel. The 10 nursing-sensitive indicators selected included three categories (American Nurses Association, 1995). See Table 1.

**Table 1:** Structure, process, and outcome indicators (American Nurses Association, 1995).

Structural Indicators	• Staff mix • Nursing care hours per patient day
Process Indicators	• Maintenance of skin integrity • Staff nurse satisfaction
Patient- focused outcome indicators	• Nosocomial infections • Patient falls • Patient satisfaction with education received • Patient satisfaction with pain management • Patient satisfaction with overall care • Patient satisfaction with nursing care

There was much debate about the sensitivity of these indicators to nursing care. It was acknowledged that these indicators were selected in part because of their sensitivity to nursing but also because of existing mechanisms for collecting data, such as the use of hospital administrative data (Gallagher and Rowell, 2003). This was the beginning of using nursing-sensitive indicators as a means of measuring nursing care.

Work continued on the development of nursing-sensitive indicators. In 2003, the National Quality Forum launched an effort to reach further consensus to define/clarify what constitutes a nursing-sensitive indicator. A total of 150 potential nursing-sensitive measures were submitted to the National Quality Forum for review. A steering committee deliberated about the strength of each measure as a consensus standard for nursing-sensitive care and its “nursing sensitivity” and, in the end, only 15 purposed nursing-sensitive indicators met the committees’ standards for endorsement (National Quality Forum, 2004). See Table 2.

**Table 2:** Structure, process, and outcome indicators (National Quality Forum, 2004).

Structure Indicators	• Skill mix • Nursing care hours per patient • Practice environment • Voluntary turnover of nursing staff
Process Indicators	• Smoking cessation counselling for acute myocardial infarction • Smoking cessation for heart failure • Smoking cessation for pneumonia
Outcome Indicators	• Mortality amongst surgical patients/ failure to rescue • Pressure ulcers • Falls • Falls with injury • Restraint use • Catheter associated urinary tract infections • Central line catheter-associated blood stream infections • Ventilator- associated pneumonia

It is worth noting that only three measures were related to nursing processes. In the early stages, there were difficulties agreeing on what aspects of care could be considered nursing-sensitive (National Quality Forum, 2004). It was debated that mortality and length of hospital stay had weak theoretical links to nursing care quality (American Nurses Association, 1995). Jones (2016) commented that these indicators were hospital-centric and, while there was a framework in place to capture all of the elements of nursing, the indicators proposed did not reflect the full scope of the nursing practice. Despite the disagreements, a substantial amount of work continued into integrating the selected nursing-sensitive indicators into healthcare practices as nursing quality measures (Alexander, 2007).

### 2.2 Current nursing-sensitive outcomes

Oner *et al*. (2021) published a systematic review of literature available on nursing-sensitive indicators from 1997 to 2017. A total of 39 studies were included, of which 74 % originated in the USA, and others were from Australia, Canada, China, and the United Kingdom (UK). The most frequently reported nursing-sensitive indicators were hospital- acquired infection, mortality, failure to rescue, patient falls, pressure ulcers development, medication administration errors, length of stay, patient satisfaction, and nurse satisfaction. It is noteworthy that some of the original indicators from the American Nurses Association and National Quality Forum are still the most frequently used (Jones, 2016). In over 20 years since the term nursing-sensitive indicator was first introduced, little progress has been made in developing its use in modern healthcare, and initial concerns raised regarding the sensitivity of indicators to nursing continue to be echoed but not addressed (Aiken *et al*., 2001; Doran, 2011; Burston, Chaboyer and Gillespie, 2014; Ryder *et al*., 2024).

## 3 Overview of the issues

Since the original development of nursing-sensitive indicators, flaws within this approach to measuring nursing care have been highlighted; however, little has been done to address the concerns (Jones, 2016). This, unfortunately, has led to lack of progress in developing a comprehensive measurement of nursing care. There has been no agreement on how quality of care should be measured and no agreed set of indicators or performance measures to capture the unique contribution that nurses make to patient outcomes (Sim et al.*,* 2018).

### 3.1 Nursing sensitive indicators as a concept

When reviewing current literature on nursing-sensitive indicators, there is a lack of consistency and standardisation of the concept. What constitutes a nursing-sensitive indicator is ambiguous, leading to different use and meanings of the term in healthcare settings (Heslop and Lu, 2014). Afaneh, Abu-Moghli, and Ahmad (2021) carried out a concept analysis on nursing-sensitive indicators and found that the terminology linked to this concept varied immensely. There was an array of terms and phrases used to depict nursing-sensitive indicators, including ‘nurse-sensitive indicator, nursing-sensitive indicator, nursing-sensitive quality indicator, nursing-sensitive outcome, nursing- sensitive outcome indicator, nursing-sensitive patient indicator, nursing-sensitive screening indicator, and nurse-sensitive outcome’. In the 36 articles included for their review, many authors did not define what they meant by nursing-sensitive indicator or include a conceptual or theoretical framework to guide their investigations (Afeneh, Abu-Moghli and Ahmad, 2021). When definitions were provided, they lacked consistency; for example, Gao et al. (2018, p.3221) refers to nursing-sensitive quality indicators as a ‘set of principle, procedure and assessment scales that are used to quantify the level of nursing quality and to assess nursing outcomes in clinical nursing practice’, whereas Stalpers et al. (2016, p.2) defined nursing-sensitive indicators as ‘outcomes that are relevant to nurses’ scope and domain of practice and for which there is empirical evidence linking nursing inputs and interventions to the outcomes for patients’. Terms were used interchangeably, even though different meanings were implied (McNett, 2018). For example, when ‘nurse-sensitive’ was used to characterise an indicator, it implied that the indicator was influenced by an individual nurse. However, in contrast, when ‘nursing-sensitive’ was used, the indicator referred to the nursing system in a broader sense (Burston, Chaboyer and Gillespie, 2014).

The concept of nursing-sensitive indicator in itself varied between studies; several sets of indicators were used without clarity on what category they fell under: structure, process, or outcome (Kieft *et al*., 2018; Murphy *et al*., 2019). Twigg (2016), used the term ‘outcome indicator’ when referring to nurse staff levels, whereas elsewhere and in keeping with the Donabedian model, nurse staffing levels were referred to as a ‘structure indicator’ (Dubois *et al*., 2017). Afaneh, Abu-Moghli, and Ahmad (2021) concluded that there was a discrepancy between the concept of nursing-sensitive indicators and commonly-used indicators. They believed that this deviation was linked to differences between the conceptual definition of nursing-sensitive indicator and the methodological development of nursing-sensitive indicators (Afeneh, Abu-Moghli and Ahmad, 2021). Heslop and Lu (2014) carried out a concept analysis of nursing-sensitive indicators reported similar findings. Both groups of researchers agreed that nursing-sensitive indicators met the criteria for a partially-mature concept only (Heslop and Lu, 2014; Afaneh, Abu-Moghli and Ahmad, 2021). This is disappointing considering that the concept of nursing-sensitive indicators is not new. As demonstrated above, what constitutes nursing-sensitive indicators is utterly confusing and could act as a deterrent to nurses hoping to use nursing indicators as an evidence base for monitoring quality of nursing care within their practice. It is not difficult to understand why nursing-sensitive indicators are not consistently used in daily nursing practice.

### 3.2 Nursing quality measures

The original purpose of nursing-sensitive indicators was to measure quality of nursing care; a panel of nursing experts was tasked with selecting nursing quality indicators (Gallagher and Rowell, 2003). However, nursing-sensitive indicators available are limited in what they tell us about quality of nursing care, as they do not measure the impact of nursing in a comprehensive way (Sim *et al*., 2018). One of the main criticisms of nursing-sensitive indicators is the focus on patient safety issues and the link between adverse patient events and nursing staff (Dubois *et al*., 2017). The majority of outcome indicators explored are limited to adverse events, such as falls, pressure sore development, medication errors, and similar events. However, these could be seen more as risk assessment as opposed to a reflection of the quality of nursing care (Kane et al., 2007). These kinds of outcome indicators are persistently reported, despite weak evidence linking them with nursing practice (Kieft *et al*., 2018), and certainly they are not an endpoint of care as outcome measurement suggests (Haj *et al*., 2013).

A substantial amount of work has continued to integrate nursing-sensitive indicators into healthcare practice as nursing quality measures in Ireland (Department of Health, 2017; Office of the Nursing and Midwifery Services Director ONMSD, & Health Service Executive HSE 2018). Considering the original purpose of nursing-sensitive indicators was to measure the ‘quality’ of nursing care, limited studies explored the relationship of nursing processes with outcome indicators. Lack of attention on process indicators was suggested as the result of difficulty analysing process due to lack of available data (Oner *et al*. 2021; Kurtzman, Dawson and Johnson, 2008). Nursing interventions are absent from most clinical administrative databases used to extract data on nursing-sensitive indicators; this also explains why patient outcomes reported on are limited to adverse events (Alexander, 2007). Without information on nursing processes, quality of care cannot be adequately assessed. Failure to invest in data collection methodologies with reliable and valid measures of nursing care processes that affect patient outcomes has resulted in an inadequate evidence base to support the nursing contribution to care (Jones, 2016).

Focusing on adverse events as outcome indicators can distract attention from other equally important elements of care (Dubois *et al*., 2017). Measurement and the reporting of an outcome draws attention to that outcome and gives it relevance and importance. Therefore, in order to have other essential outcomes, such as health promotion and alleviation of suffering, acknowledged, nurses must advocate for them and ensure that they are also measured (Kitson *et al*., 2013). It would be an enormous loss if aspects such as these were not recognised or invested in (Jones, 2016). Not giving all aspects of care equal importance sets the precedent of what nurses might concentrate on, as nurses are more likely to prioritise care that gets measured or audited; this could result in a patient's individual needs being overlooked (Doran, 2011). Therefore, it is reasonable to suggest that the original purpose of using nursing-sensitive indicators, which was to improve quality of care, has been lost sight of (Sim *et al*., 2018). Perhaps an element of convenience when selecting the original indicators has had lasting repercussions for the use of nursing-sensitive indicators in today's healthcare systems.

### 3.3 Fundamentals of nursing care

Current nursing-sensitive indicators do not address fundamental nursing care (Mainz, Odgaard and Kristensen, 2023), which is recognised as the foundation of nursing practice (Kitson *et al*., 2019). Historically the idea of care has been epitomised by nursing, synonymous with the image of Florence Nightingale caring for and comforting soldiers during the Crimean war (Straughair, 2012). Nightingale emphasised the importance of sanitation and nutrition, basic human needs that an individual would carry out for themselves if able. Based on descriptions of nursing from this time, a link was established between nursing care and self-care, which is consistent with Virginia Henderson's definition; ‘The unique function of the nurse is to assist the individual, sick or well, in the performance of those activities contributing to health or its recovery (or to peaceful death) that he would perform unaided if he had the necessary strength, will or knowledge and to do this in such a way as to help gain independence as rapidly as possible’ (Kitson, 1999, p.43).

The work of Roper, Logan, and Tierney (2001) provided a substantial contribution to the understanding of the activities of daily living as the core elements of self-care that require nursing intervention. These became known as the fundamentals of care, a set of universal activities essential for life. All 12 activities include the following: maintaining a safe environment, communication, breathing, eating and drinking, eliminating, personal cleansing and dressing, controlling body temperature, mobilising, working and playing, expressing sexuality, sleeping, and dying (Roper, Logan and Tierney, 2001). Nurses historically and currently have a recognised responsibility to manage these processes, regardless of a person's clinical condition or the setting (Kitson et al.*,* 2010). However, these self-caring tasks that nurses are apparently responsible for are not well defined, and this has, unfortunately, resulted in a long-standing difficulty between the written theory and the actual practice of nursing (Ottonello et al., 2023; Richards et al.*,* 2018).

Despite the integral association between nursing and fundamental care, there is a substantial void of nursing-sensitive indicators that evaluate fundamental nursing care. This is partly related to the lack of any empirical data on fundamental nursing care. Richards *et al*. (2018) reviewed the evidence for nursing care interventions associated with nutrition, elimination, mobility, and hygiene, four aspects historically noted as fundamental (Kitson, 1999). They concluded that evidence was sparse, of poor quality, and unfit to provide evidence-based guidelines to practicing nurses (Richards *et al*., 2018). They noted that not one nursing intervention stood out as clear or evidence- based. Without evidence, nurses cannot definitively demonstrate that fundamental care is being carried out or achieved.

### 3.4 Nursing values

Current nursing-sensitive indicators available are not designed to capture nursing values (Moran, Bailey and Doody, 2021). Following reports of poor care quality and apparent lack of compassion, values of the nursing profession have been revisited (Francis, 2013; Department of Health, 2012). Values are defined as a belief; beliefs can be influenced by many factors, including knowledge, experience, upbringing, culture, and religion (Baillie and Black, 2015). Different countries have adapted different sets of values through means of consultation and consensus with nurses themselves (Ballie, 2017). The UK has implemented the 6Cs: care, compassion, competence, communication, courage, and commitment (Department of Health, 2012), whereas, in Ireland, care, compassion, and commitment have been identified by nurses as the core values that underpin their profession (Nursing and Midwifery Board of Ireland, 2021). There is an expectation that nurses and midwives be competent and safety conscious and act with kindness and compassion to provide safe, high-quality care.

The Code of Professional Practice in Ireland states that nurses and midwives are morally accountable for upholding key nursing values (Nursing and Midwifery Board of Ireland, 2021). Nurses are required to incorporate their values and evidence-based clinical judgement to achieve quality and safe nursing practice. While in theory, this sounds rational, clear, and logical, there remains no means of ensuring that the Code of Professional Practice is implemented in practice. On the contrary, there is more evidence to suggest that nursing values are poorly adhered to (Department of Health, 2016; McSherry *et al*., 2018). The Health Service Executive and Nursing and Midwifery Board of Ireland have committed to supporting the integrative type of care as called for in the Code by means of employment, managerial processes, education, and regulatory processes (Department of Health, 2016). While there are documents and guidelines available, the Code of Professional Conduct and Ethics for Registered Nurses and Registered Midwives and Scope of Nursing and Midwifery Practice Framework (Nursing and Midwifery Board of Ireland, 2021; Nursing and Midwifery Board of Ireland, 2015), it is difficult to decipher if and how these documents have influenced nurses providing daily clinical care. Despite the commitment of the healthcare service to person-centred care (Department of Health, 2017), there continues to be a mismatch between policy, nursing theory, and the patient's experience in the healthcare setting (Muntlin *et al*., 2023).

Values influence attitudes and professional behaviour, including how we prioritise care and the quality of care provided. They are influenced not only by an individual but also by the organisational culture (Ballie, 2017). We must have an understanding of the values and beliefs of those we care for; in this way, we gain an understanding of the person as an individual with a unique set of needs (Kitson et al.*,* 2013; Maben et al., 2007). It is referred to as person-centred care, which has become the cornerstone of quality healthcare in many developed countries, where it is referenced in many healthcare policies (Department of Health and Human Services, 2012; New South Wales Department of Health, 2009; Government of South Australia, 2015). Person-centred care focuses on healthcare that involves patients via greater decision- making and choice and that is sensitive to patients’ unique physical, psychosocial, cultural, and emotional needs (Avallin *et al*., 2023; Kitson et al.*,* 2013). Meeting a person's intimate care needs requires focusing on and connecting with that person, understanding their needs, and interacting with them in a way that they feel safe, valued, and understood (Kitson et al.*,* 2013). Developing the type of nurse-patient relationship where this kind of caring can occur is not a simple task-based exercise (Jackson and Kozolowska, 2018). Nurses cannot provide fundamental care to a patient without upholding their professional values.

## 4 Discussion

It is clear from the issues outlined above that many of the current nursing-sensitive indicators available are not sufficient to evaluate the quality of nursing care, yet little progress has been made developing an alternative method (Sim *et al*., 2018). Many researchers have examined nursing indicators (Griffiths *et al*., 2018; Twigg *et al*., 2016; Gao et al.*,* 2018; McNett, 2018), but most of them have focused on particular aspects of nursing care in specific settings, and, therefore, their conclusions cannot be used as a comprehensive guide for general nursing practice. Researchers primarily use structure indicators (staffing numbers, skills mix) or outcome indicators (urinary tract infections, pressure sores, falls); however, process indicators (mobilisation, nutritional support, hygiene) are rarely reported on (Mainz, Odgaard and Kristensen, 2023). However, it is the non-fulfilment of process indicators that is often referred to as missed care, leading to some of the alarming finding of poor care in healthcare settings (Francis, 2013; McSherry et al., 2018; White, Aiken and McHugh, 2019).

In modern healthcare, there is a focus on throughput and measurement of efficiency. This has accumulated in numerous methods to measure nursing activity, such as nursing metrics, minimum data sets, and electronic records of outcomes. Systems such as these leave little room for examining fundamental care provided (Pentecost *et al*., 2020). Emphasis is misplaced on task completion and outcomes evaluation in order to prioritise cost, productivity, and efficiency (Griffiths et al., 2018). Tadd *et al*. (2011) found that there was often undue emphasis on the recording of care but not how that care was actually delivered; this could be seen as a form of metric-based harm (Terry, 2016). In a system such as this, nurses are encouraged to concentrate on technical and physical work that can be objectively measured (Bridges et al.*,* 2013). This type of healthcare care is depersonalised and mechanistic, with no emphasis on meaningful engagement with people to deliver personalised care (Kitson et al.*,* 2019; Maben et al., 2007). For example, preventing falls does not always equate to good nursing care, as concluded by Groves et al. (2017), where inappropriate use of physical restraint was linked to violation of patient's independence. This is the very opposite of what nurses should be trying to achieve, which is promoting patient independence. The findings in the Francis (2013) report are a product of a healthcare system that prioritises meeting metric driven targets as opposed to providing person-centred care.

### 4.1 Metrics

Nursing metrics are agreed standards of measurement for nursing and midwifery care, where care can be monitored against agreed standards and benchmarks (Foulkes, 2011). Ireland has adapted a National Guideline for nursing and midwifery quality care-metrics data measurement (Office of the Nursing and Midwifery Services Director ONMSD, & Health Service Executive HSE 2018). Quality care-metrics aim to provide nurses and midwives with a framework and measurement tool to engage with continuous quality improvement at the point of care delivery (Murphy *et al*., 2019). The Office of the Nursing and Midwifery Services Director is responsible for leading the national implementation of nursing and midwifery quality care metrics in Ireland. The concept arose from work undertaken in the UK by the Heart of England National Health Service Foundation Trust. A web-based tool entitled Test Your Care was developed by nurses. Data is collected from care records and a traffic light system is used for scoring, based on information included on various nursing assessments. A list of validated nursing assessment tools has been provided for use compatible with the system (Office of the Nursing and Midwifery Services Director ONMSD, & Health Service Executive HSE 2018). While this system provides information on nursing assessment and metrics on adverse outcomes, such as falls, rates of infection, pressure sore development, and similar outcomes, it fails to acknowledge the caring processes involved or the quality of care delivered. There is continued lack of recognition of the fundamental care processes and how they lead to positive patient outcomes (Feo, Kitson, and Conroy, 2018).

It was hoped that once data was collected on quality care-metrics, it could then be presented as performance indicators for nursing in Ireland (Department of Health, 2017). This is referred to in the Framework for National Performance Indicators. The Irish health sector wanted to evaluate the contribution of nurses and midwives to healthcare and introduce a greater sense of accountability for nurses. They believed the metrics would guide the government not only on areas that needed more support but also on the areas that were performing well within in the nursing workforce (Department of Health, 2017). However, nurses need to ask how can they allow themselves to be judged, evaluated, and subsequently guided by a set of metrics that doesn't acknowledge the bedrock of their practice; providing patient-centred fundamental care to achieve positive patient outcomes (Moran, Bailey and Doody, 2021).

### 4.2 Classification of nursing terminology

To standardise the terminology of nursing practice, international researchers and organisations have developed a range of individual models (Hannah et al.*,* 2012). The International Council of Nurses has developed an International Classification for Nursing Practice to systematically describe nursing; other models include the North American Nurses Diagnosis Association (NANDA), which developed the Nursing Interventions Classification (NIC) and the Nursing Outcomes Classification (NOC) (Rabelo-Silva and Cavalcanti, 2017). Nursing Outcome Classification was introduced by the University of Iowa and is one of the standard classification systems widely used for outcome selection in nursing care plans (Moorhead et al.*,* 2018). Classifications of this nature have the potential to enhance communication between healthcare settings and professionals and are used to develop measures for comparison and statistical reports to influence policy and nursing practice (Rodríguez- Suárez *et al*., 2023). Electronic healthcare records and the development of patient registries bring about opportunities to sample big data (Dochterman et al.*,* 2018). The standardised classifications might add some valuable information to assessment of nursing practice but should be used with caution (Othman et al., 2020; Muntlin, 2018). Current classification data, such as nursing diagnosis, interventions, and outcomes found in the International Classification for Nursing Practice, Nursing Interventions Classification, and Nursing Outcomes Classification do not fully address a person-centred approach to care; for instance, a nursing diagnosis can be given a code with a specific set of related interventions, leaving no room for individualised care and the patient-centred approach advocated internationally (Kitson, 2018; Bartz, 2010).

### 4.3 Lack of focus on fundaments and values

Kitson *et al*. (2010) voiced concern about how fundamental care is perceived as a marginal contributor to patient outcomes that can be delivered by less-skilled staff than nurses. This attitude has reduced formal measurement and evaluation of fundamental care, resulting in lack of evidence in delivery and outcome of implementing fundamental care (Kitson *et al*., 2019). The worrying fact is that as a profession we do not actually know what skills are necessary to deliver and manage fundamental care (Ryder *et al*., 2022). Effort to refocus nursing on fundamental care revealed the lack of evidence to guide nursing practices in this area (Richards *et al*., 2018; Pentecost *et al*., 2020; Ryder et al. 2022). The evidence for fundamental care nursing interventions is sparse and nowhere near sufficient to provide the evidence-based guidelines required (Pentecost et al.*,* 2020). It is difficult to comprehend that while these elements have been described as ‘essential’ to nursing, they are not documented or defined in any concise way (Feo et al.*,* 2019). There is minimal understanding of how nurses’ fundamental care, such as patient mobilisation, differs or intersects with that of other healthcare professionals, compared with the physiotherapist or rehabilitation doctor. How does the nurse's daily engagement with the patient link to the recovery goal (Feo, Kitson and Conroy, 2018)? Richards *et al*. (2018) harshly commented that, in the absence of evidence, nurses conducted their duties using a combination of guesswork, folk knowledge, and tradition or else using evidence from studies in which the findings were weak.

Failure to value fundamental skills enough to research them in detail is problematic for nurses as they have the over-all responsibility for this care. Kitson et al. (2010) questioned whether it is not as important to research the most effective way of encouraging elderly patients suffering from dementia to chew and swallow their meals as it is to research gastric emptying in elderly patients suffering from hypotension after mealtimes (Kitson *et al*., 2010). The notion that fundamental care doesn't require skills to execute has led to fundamental care been delegated to other care staff, such as healthcare assistants (Health Service Executive, 2018), while experienced and educated nurses focus on more technical tasks, such as wound care and medication administration (Wolf, 2014). There is also a blame on so-called ‘academic nursing’ for the short comings in patient care. Some believe that the academic development of nursing has removed compassion from the role (Hayter, 2013; Richardson, Percy and Hughes, 2015). In an era of modernised and professional nursing, there appears to have been an oversight in the core values of nursing and what is central to patient care (Mudd *et al*., 2020).

Lack of direction and leadership in fundamental care has resulted in nurses feeling powerless in care‐related decisions (Kneafsey et al.*,* 2013; Robison *et al*., 2014) Absence of clear responsibilities has led to confusion and uncertainty for nurses, even when making minor decisions, such as which incontinence aids to use for patients (Taylor *et al*., 2014a). Due to limited resources, nurses often feel under pressure to prioritise their work, and often it is fundamental care that is left undone (Muntlin et al.*,* 2023). Nurses themselves have reported that due to time constraints, fundamental care, such as oral care, comforting, and educating patients and their families, is not carried out (Aiken *et al*., 2014; Ball et al., 2014). Nurses often feel challenged in the current healthcare system, as it does not recognise or reward caring (Aiken *et al*., 2014). Engaging in work that is not valued or feeling unable to provide the type of care that is fundamental can be morally distressing (Bridges *et al*., 2013). This may explain that, while many nurses enter the nursing profession with a desire to provide high quality fundamental care, they often end up leaving or getting burnt out in the task-based environment (Muntlin, 2018). Perhaps disengagement from patients and devaluing of fundamental care is a coping mechanism and a means of self-preservation that nurses have adapted to survive in the current healthcare system (Bridges *et al*., 2013).

### 4.4 Current work to address the issues

There is an international campaign to identify and incorporate fundamental care into the assessment of the quality of nursing care (Kitson *et al*., 2010; 2018; Feo *et al*., 2019). The International Learning Collaboration recognised the consequences of not addressing core values in nursing and set about outlining a framework to implement fundamental care in a way that is relevant to clinical practice. Members of the International Learning Collaboration wanted to refocus nursing on its founding principles of care and compassion for individuals, while also achieving physical outcomes (Feo et al.*,* 2019). The collaboration was first established in 2008 by nurse leaders with clinical experience and expertise who continue to work on application of the framework (Kitson, 2018). It is inevitable that the clinical condition of a person will affect the delivery of fundamental care; however, the International Learning Collaboration emphasise that safe, person-centred care needs to be achieved through addressing a patient's physical, psychosocial, and relational needs (Feo and Kitson, 2016). A conceptual framework outlined by the International Learning Collaboration recognises three distinct dimensions of care: the relationship, the integration of care, and the care context (International Learning Collaboration, 2022). The International Learning Collaboration group's work is on-going and includes innovative methods, such as the use of complexity science as a means of measuring fundamental care (Conroy *et al*., 2023). Unfortunately, there remains no definitive means of measuring the impact of this holistic type of care on patient outcomes, and few healthcare systems are using the framework in their daily practice (Avallin et al.*,* 2023). However, it cannot be accepted that it is simply too difficult to measure quality nursing care, and work to achieve this must be supported at national and international level (Richards et al.*,* 2018).

Some healthcare organisations have created fundamental care standards and initiatives (Care Quality Commission, 2011; New South Wales Department of Health, 2009); however, some of these government documents were produced in response to a specific concern about care delivery in a particular area. For example, a New South Wales government document that focused on patient dignity and respect was published in response to complaints about neglect of these aspects of care (New South Wales Department of Health, 2009). Others, such as Carr and Benoit (2009) and Vollman (2013), have also focused their research on specific fundamental care interventions, such as such as hygiene or oral care. While these initiatives are important and a step in the right direction, they lack a comprehensive view of the fundamentals of care in practice (Burston, Chaboyer and Gillespie, 2014). The efforts are arguably disjointed, and nurses can't see a the benefit or impact of such initiatives in their daily routine. Multiple initiatives have led to chronic fatigue within the nursing work force, as there is a constant bombardment of apparently- random initiatives, related to isolated aspects of care and yet more paper-based exercises (Kitson *et al*., 2018).

## 5 Conclusion

Despite what is known about the weak theoretical links between nursing care quality and current nursing-sensitive indicators, many of the same historic nursing-sensitive indicators are used over and over again as measures of quality nursing (Terry, 2016; Aiken *et al*., 2014; Doran, 2011). Failure to address fundamental nursing care and values in the use of nursing-sensitive indicators has manifested as repeated findings of poor patient care (Garling, 2008; Willis Commission, 2012; Francis, 2013; Groves *et al*., 2017).

Nurses are better equipped than ever (through education, training, and application of technology) to highlight the true quality of their care. There is urgent need for a set of nursing-sensitive indicators that truly reflects fundamental nursing care, and this should be the minimum standard, rather than the exception, across healthcare systems (Kitson et al.*,* 2019). The International Learning Collaboration continues to lead the way on the integration of fundamental care through development and use of their framework in practice (International Learning Collaboration, 2022; Avallin *et al*., 2023). However, it is the responsibility of nurse educators and nurse leaders to take ownership of the evaluation of person-centred evidence-based fundamental care (Hakami et al.*,* 2023); otherwise, policy makers and academics will continue to measure the quality of nursing care through inappropriate means. Researchers must be willing to go beyond secondary analysis of hospital databases (Jones, 2016). Nursing quality assessment and nursing-sensitive indicators research initiatives have historically been hindered by lack of available data related to the nursing process (Doran, 2011). It is crucial that nurses take time to consider the future of quality nursing care and how this can be demonstrated and protected within the ever demanding healthcare system.

## Recommendations

• Practice: Nurses need to be included in discussions regarding nursing-sensitive indicator research at practice level. They need to be involved in reaching a consensus on how nursing care can be monitored and evaluated best; this must be encouraged, supported, and guided by nursing management. Quality nursing care needs to be valued at the healthcare system level, so that appropriate resources can be dedicated to achieving valid nursing-sensitive indicators.
• Research: Proposed nursing-sensitive indicators need to be researched and validated to ensure they are capturing nursing care sufficiently. Studies need to be standardised and repeated across various healthcare facilities until the selected nursing-sensitive indicators are deemed reliable and accurate for use.
• Education: Continuous professional development and education is central to nursing. The importance of evaluating nursing care needs to be incorporated into nursing education at undergraduate and post-graduate levels. Nursing-sensitive indicators should be discussed in an environment that encourages the sharing of knowledge and experience, where both are valued, and used to formulate accurate methods of evaluating care. An understand of the nurse's integral role in patient care should be fostered, and nurses should be able to recognise the impact of their work through the use of appropriate nursing-sensitive indicators.

## CRediT authorship contribution statement

**Edel Gormley:** Writing – review & editing, Writing – original draft. **Michael Connolly:** Writing – review & editing. **Mary Ryder:** Writing – review & editing.

## Declaration of competing interest

The authors declare that they have no known competing financial interests or personal relationships that could have appeared to influence the work reported in this paper.
